# A Glass Fiber-Reinforced Resin Composite Splint to Stabilize and Replace Teeth in a Periodontally Compromised Patient

**DOI:** 10.1155/2020/8886418

**Published:** 2020-07-21

**Authors:** Angelika Rauch, Sebastian Mehlhorn, Manja Mühle, Dirk Ziebolz

**Affiliations:** ^1^Department of Prosthodontics and Materials Science, University of Leipzig, Liebigstr. 12, 04103 Leipzig, Germany; ^2^Private Practice, Praxis Dietrich Mehlhorn, Tannenbergsthaler Str. 7, 08269 Muldenhammer, Germany; ^3^Department of Cariology, Endodontology and Periodontology, University of Leipzig, Liebigstr. 12, 04103 Leipzig, Germany

## Abstract

Combined periodontal and prosthodontic treatment demands of patients require a structured coordination of pretreatments and an adequate choice of restorations. This is especially true if multiple teeth are missing and insufficient crown-root ratios are obvious. A 40-year-old patient with a severe periodontitis (Stage IV, Grade C) was treated with active, nonsurgical periodontal therapy. Afterwards, a supportive periodontal therapy was provided with a recall interval of three to four months. Due to a high tooth mobility of the anterior teeth in the upper jaw and a missing left canine, a combination of a resin composite (Signum composite, Kulzer, Hanau, Germany) and a unidirectional glass prepreg fiber (Tender Fiber Quattro, MICERIUM, Avegno, Italy) was utilized to fabricate a splint in a labside approach to stabilize the remaining teeth. Moreover, an artificial denture tooth was adhesively luted to the splint. A temporary polymer-based material (Vita CAD-Temp, VITA Zahnfabrik, Bad Säckingen, Germany) was selected to supply the posterior teeth of the patient with a 3-unit fixed dental prosthesis (FDP), and both restorations were adhesively cemented. 19 months after insertion, a fracture of the fiber-reinforced resin composite splint occurred that was intraorally repaired. In spite of the fracture of the splint, all materials were functionally and esthetically stable over the follow-up period of 22 months.

## 1. Introduction

Due to their complexity, patients with both periodontal and prosthodontic treatment demands are challenging as coordination of pretreatments and adequate selection of restoration type and material is required. In patients with periodontal bone loss characterized by multiple missing teeth and/or insufficient crown-root ratios, removable partial dentures (RPDs) might be efficient. Nonetheless, abutment teeth of RPDs, especially in function of direct retainer elements, present a higher risk of tooth loss in combination with a periodontal disease in comparison to nonabutment teeth [1–4]. For multiunit fixed dental prostheses (FDPs), loss of abutment teeth is assumed to be less than for RPDs [1]; yet to supply a patient with an FDP, the removal of tooth hard tissue is necessary. For FDPs in periodontally compromised patients, long-term temporary polymer-based materials are preferably used as they are cost-efficient. Polymer-based materials can be processed in press technique or fabricated with computer-aided design/computer-aided manufacturing (CAD/CAM). Recent investigations revealed that CAD/CAM-fabricated polymer-based materials are superior to conventionally pressed restorations since lower biofilm formation can be expected [5].

Advanced bone loss in periodontally compromised patients often increases the mobility of teeth, which can indicate the splinting of the residual teeth. As side effect, this measure reduces the risk of traumatic dynamic occlusion [6]. Teeth can be splinted with fixed or removable devices, as temporary or permanent therapy strategy. Therefore, fibers are frequently chosen, which can be fabricated from different materials such as of polyethylene or glass [7]. A pilot study investigated glass fiber-reinforced composite resin splints that were directly applicated in patients. The research group observed high survival rates and favorable periodontal status of the splinted teeth over a timeframe of four years [8]. These results are corroborated by long-term results revealing that splinted teeth are not affected by an increased risk of tooth loss in patients with periodontitis [9]. Moreover, splinting procedures can instead induce bone remodeling processes that prevent bone loss [10].

The aim of this paper was to report about the treatment of a patient with severe periodontitis (Stage IV, Grade C) and a need for prosthetic treatment. An interdisciplinary concept was established that included active, nonsurgical and a consequent supportive periodontal therapy (SPT). A resin composite splint that had been stabilized by a glass prepreg fiber was adhesively luted to an artificial denture tooth and a 3-unit FDP made out of a temporary polymer-based CAD/CAM material were fabricated and adhesively cemented. A follow-up over a period of 22 months was conducted. This case report describes the use and short-term clinical outcome of a fiber-reinforced resin composite splint and an FDP fabricated from a temporary polymer-based CAD/CAM material in a periodontally compromised patient.

## 2. Case Presentation

**Figure 1:**
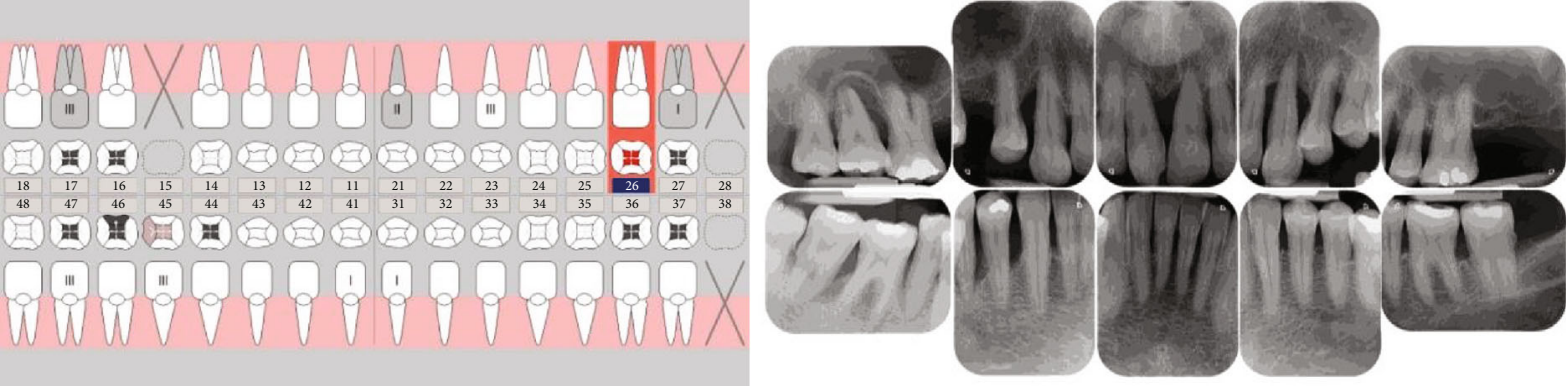
Results of the dental examination (12/2014).

**Figure 2:**
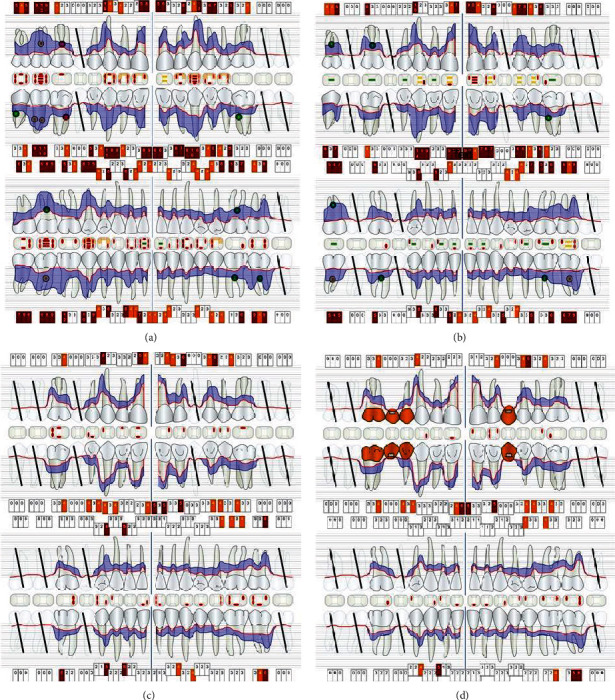
Periodontal status of the patient (a) in May 2015, (b) before (01/2016) and (c) after active, nonsurgical periodontal treatment (10/2016), and (c) at follow-up (10/2019).

**Figure 3:**
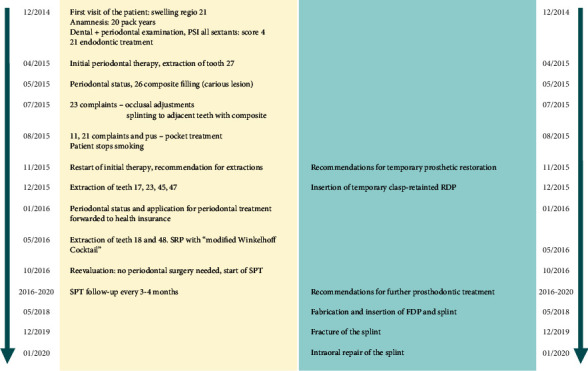
Time scale of the periodontal (left) and prosthodontic treatment (right).

A 40-year-old male patient presented to the dental ambulance of the University of Leipzig due to complaints and a gingival swelling in the area of the upper anterior teeth in December 2014. The patient was healthy and no medication was notated. He stated to smoke twenty cigarettes a day (20 pack years). The examination of the extraoral facial area and the oral mucosa revealed no abnormalities; the intraoral dental and X-ray status are presented in Figure 1. The periodontal screening and recording index (PSR index) was measured revealing scores of four in every sextant (minimum one periodontal probing depth (PPD) > 5.5 mm). The initial active periodontal therapy as well as endodontic treatment was initiated (Figures 2(a) and 3).

**Figure 4:**
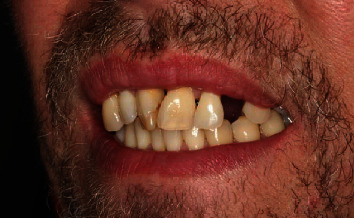
Esthetical appearance of the patient after extraction of tooth 23.

Nonetheless, within the following months, the patient showed up irregularly and only symptom-orientated. Tooth 27 was extracted due to a combined endo-perio lesion. In July and August 2015, the patient complained about problems with the anterior teeth of the maxilla. Adjustments of the occlusion, splinting of tooth 23, and pocket treatments were conducted. The patient quitted smoking. In November 2015, initial periodontal therapy was started again, which included several professional tooth cleaning appointments (including oral health instruction and motivation) and extractions of teeth 18, 17, 23, 45, 47, and 48. To compensate the esthetical shortcomings after loss of the canine (Figure 4), the patient was supplied with a temporary RPD. The diagnosis after measurements for the periodontal status was severe periodontitis (Stage IV, Grade C, Figure 2(b)). The active, nonsurgical periodontal treatment with hand instruments, oscillated ultrasonic scaler (Cavitron Select SPS, Dentsply Sirona, York, Pennsylvania, USA), and “*modified Winkelhoff Cocktail*,” i.e., 3 × 500 mg amoxicillin plus 3 × 400 mg metronidazole for 7 days, was applied in May 2016. During several follow-ups scheduled in a range of seven days, three weeks, three months, and five months, no complaints occurred and the periodontal parameters (PPD and attachment loss (AL)) improved (Figure 2(c)). Thus, no periodontal surgery was initiated, and the patient received SPT with a follow-up interval of three to four months. Further prosthodontic treatment was planned.

**Figure 5:**
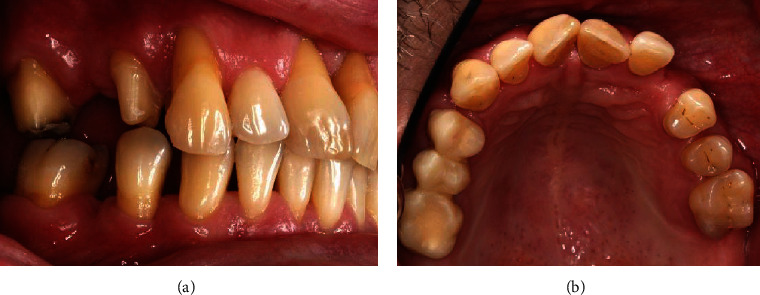
Preparation design of the abutment teeth for the (a) 3-unit FDP and (b) fiber-reinforced resin composite splint.

**Figure 6:**
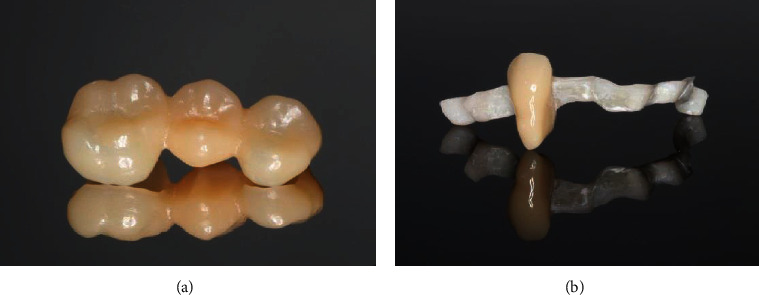
The (a) 3-unit FDP and (b) fiber-reinforced resin composite splint after fabrication.

**Figure 7:**
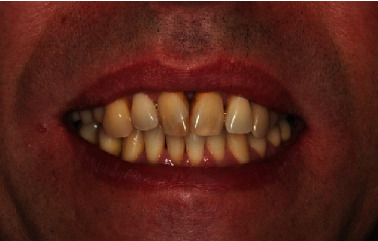
Esthetical appearance of the patient after insertion.

In view of the mandibula, no need for prosthetic treatment was identified, as tooth 46 had nearly closed the gap of the extracted tooth 45. To solve the situation in the maxilla, implantological measures were suggested but financial reasons and the need for bone augmentation made the patient deny implantology. The low tooth mobility and the acceptable crown-root ratios of the posterior teeth indicated the fabrication of a 3-unit FDP to replace tooth 15. Nonetheless, the unfavorable crown-root ratios of the anterior teeth as well as the healthy hard tissue of teeth 22 and 24 were reasons to withdraw the idea of another FDP. Moreover, the tooth mobility of the anterior teeth was high. Thus, in agreement with the patient, we decided to fabricate a 3-unit FDP replacing tooth 15 as well as a splint covering teeth 12-25. In addition, the splint was supposed to replace tooth 23. The FDP should be made out of a temporary polymer-based restorative CAD/CAM material (Vita CAD-Temp, VITA Zahnfabrik, Bad Säckingen, Germany), which is available for long-term use. For the fabrication of the splint, a combination of a glass prepreg fiber (Tender Fiber Quattro, MICERIUM, Avegno, Italy) and a resin composite (Signum composite, Kulzer, Hanau, Germany) should be chosen, which could be adhesively luted to an artificial denture tooth (VITAPAN PLUS, VITA Zahnfabrik). In May 2018, the preparation of the 3-unit FDP was realized in accordance with the guidelines of the manufacturer and the abutment teeth of the splint were prepared slightly, only touching the enamel, to ensure the same direction of insertion (Figures 5(a) and 5(b)). Afterwards, a conventional impression was taken using a polyvinyl siloxane impression material (Aquasil Ultra Heavy and Aquasil Ultra+ XLV, Dentsply Sirona) and the restorations were fabricated labside (Figures 6(a) and 6(b)). The try-in procedure of the FDP and the splint revealed that the patient was pleased with oral comfort and his esthetical appearance (Figure 7). After sandblasting, both restorations were adhesively cemented (Celtra Cementation System, Dentsply Sirona).

**Figure 8:**
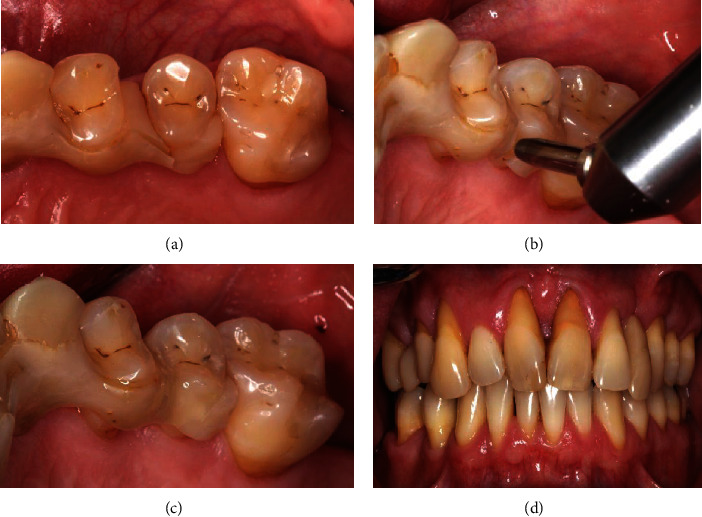
At follow-up, a fracture of the splint was observed in regio 24/25 (a), which was repaired utilizing sandblasting, a universal primer, and a direct composite (b–c), follow-up (d).

Afterwards, SPT follow-up was continued as described above, which included a reevaluation of the periodontal parameters (PPD and AL) in October 2019. A stability of periodontal conditions with signs of gingivitis based on severe periodontitis (Stage IV, Grade C) was observed (Figure 2(d)). In December 2019, a fracture of the splint in regio 24/25 was examined (Figure 8(a)). For repairing purposes, the fractured parts were slightly removed with a bur and roughened by using sandblasting (CoJet, 3M, Seefeld, Germany) (Figure 8(b)). The enamel was etched with 35% orthophosphoric acid (Vococid, VOCO, Cuxhaven, Germany) for 30 seconds and a universal primer (Scotchbond Universal, 3M) was applied for 20 seconds to the roughened areas of the splint and to the enamel. Afterwards, a direct resin composite (ceram.x, Dentsply Sirona) was used to connect the fractured ends of the splint (Figure 8(c)). At SPT follow-up appointment in March 2020, stable periodontal conditions were identified. The patient did not describe any complaints or complications (Figure 8(d)).

## 3. Discussion

The results of this case report emphasize that for patients with complex periodontal and prosthetic treatment demands an interdisciplinary approach might be necessary as a coordination of pretreatments and adequate prosthetic interventions is required. Moreover, the application of innovative materials and techniques can be helpful to solve challenging dental situations. In spite of the fracture of the splint, the applied materials were functionally and esthetically stable over a follow-up period of 22 months.

Within the literature, very high complication rates of splinted teeth are described. The frequency of repairs was approximately 75% and nearly half of the splints needed to be repaired annually [9, 11]. The risk factors for technical events seem to be associated with the severity of bone loss and the location of the splinted teeth, as complications are less likely in the anterior teeth of the mandibula but even more likely in the posterior teeth [9]. These suggestions can be corroborated by the results of this case report, as high bone loss was associated with the splinted teeth. Moreover, the fracture of the splint occurred in regio 24/25, an area that withstands high occlusal forces. Consequently, stress onto the splint might have been high. In view of the survival of the FDP, a prospective study examined 3- to 4-unit FDPs fabricated from the same temporary CAD/CAM polymer-based material (Vita CAD-Temp, VITA Zahnfabrik) as presented in our case report. Depending on the design of the restorations, excellent short-time survival rates were observed if a terminal abutment design was chosen; however, cantilevered FDPs presented increased complication rates [12]. For the prosthetic therapy of periodontally compromised patients, the temporary polymer-based CAD/CAM material might be an interesting option, as a recent investigation described its low association with biofilm formation. The study group examined colonies consisting of *Streptococcus sanguinis*, *Fusobacterium nucleatum*, and *Porphyromonas gingivalis* but observed that biofilm was statistically significantly less attached to the temporary polymer-based CAD/CAM material than to 3 mol % yttria-stabilized tetragonal zirconia ceramics or polymer-infiltrated ceramic network materials [13].

In general, the present casuistic emphasizes the importance of patient compliance and maintenance in both periodontal and prosthodontic therapy. Recent investigations concluded that tooth loss, tooth mobility, and other complications in patients with RPDs might be decreased by suitable supportive care and a scheduled follow-up of at least six months [14–16]. Even for fixed restorations, appropriate instructions, periodontal maintenance, and self-motivation for plaque control are essential to avoid negative effects on the periodontium [17, 18].

The results of this case report might help to solve difficult prosthodontic cases in periodontally compromised patients and might push dentists to think “outside the box.” Further research on mechanical properties and clinical behavior of labside fabricated splints for periodontal purposes will be needed to better estimate the effect on tooth- or splint-related outcomes.
